# Bipolar versus monopolar resection of benign prostate hyperplasia: a comparison of plasma electrolytes, hemoglobin and TUR syndrome

**DOI:** 10.1186/s40064-016-3407-7

**Published:** 2016-10-07

**Authors:** Meltem Savran Karadeniz, Erdem Bayazit, Omur Aksoy, Emine Aysu Salviz, Tzevat Tefik, Oner Sanli, Mukadder Orhan Sungur, Kamil Mehmet Tugrul

**Affiliations:** 1 Department of Anesthesiology, Medical Faculty of Istanbul, Istanbul University, Istanbul Universitesi Tip Fakultesi, Anesteziyoloji AD, Millet Cad. Cerrahi Monoblok Giris Kati, Fatih, 34093 Istanbul, Turkey; 2 Department of Urology, Istanbul Faculty of Medicine, Istanbul University, Istanbul, Turkey

**Keywords:** Transurethral resection of prostate, Monopolar, Bipolar, 5 % Mannitol, 0.9 % Sodium chloride, Serum electrolytes

## Abstract

**Background:**

Bipolar and monopolar transurethral resection of prostate (TURP) are both widely used for surgical treatment of benign prostatic hyperplasia. Systemic absorption of irrigation fluids during TURP operations leads to variations in blood chemistry. The aim of this prospective clinical study was to compare two different surgical techniques and the systemic effects of irrigation solutions (5 % mannitol vs. 0.9 % sodium chloride) under standardized anesthesia care.

**Methods:**

Fifty-two patients who were scheduled for elective TURP were enrolled in the study. Patients were divided into two groups; the group M; 5 % mannitol was used for irrigation and the group B; 0.9 % sodium chloride was used for irrigation. Spinal anesthesia was performed to all patients. The patients’ demographics, prostate volumes, hemodynamic parameters, volumes of irrigation, and IV fluids were recorded. Serum electrolytes (Na^+^, K^+^) and hemoglobin (Hb) were analyzed in blood samples taken before the operation (control), at the 45th min of the operation (1st measurement), and 1 h after the end of the surgery (2nd measurement) and recorded.

**Results:**

The Na^+^ value of group M was significantly lower in both the 1st and 2nd measurements compared with the control value (*p* < 0.001 and *p* < 0.001). Na^+^ values of group M were also significantly lower than group B in both the 1st and 2nd measurements (*p* < 0.001 and *p* < 0.001). The change in Na^+^ levels was found to be statistically significant (*p* < 0.001) in group M, whereas the intergroup changes were not statistically significant in group B.

**Conclusion:**

Our results demonstrated that bipolar resection coupled with 0.9 % sodium chloride has minimal effects on serum sodium levels compared with monopolar resection.

*Clinicaltrials.gov identifier* NCT02681471

## Background

Benign prostatic hyperplasia (BPH) is a common pathology seen in elderly man. Medical management, transurethral resection of the prostate (TURP), and open prostatectomy are the treatment options. TURP is accepted as the gold standard for the surgical treatment of BPH in appropriate patients because it is less invasive compared with the open technique and has lower complication incidence (Reich et al. 2006; De la Rosette et al. 2001). Monopolar or bipolar resection techniques have been used to resect prostatic tissue. In monopolar resection, systemic absorption of the electrolyte-free irrigation solutions (i.e. glycine, sorbitol, and mannitol) and high-frequency electrical current may cause complications such as incontinence; erectile dysfunction; stenosis of the bladder neck; bleeding; and transurethral resection (TUR) syndrome (2 %). TUR syndrome is caused by absorption of high volumes of electrolyte-free irrigation solution to the systemic circulation (Norlen 1985).

Introduction of bipolar resection to clinical practice enabled surgeons to work with isotonic saline as an irrigation solution and low-voltage current, which made resection and cauterization possible at the same time. Bipolar resection also ensures better vision quality, less tissue damage compared with the monopolar resection, and protects against TURP syndrome. However, fluid overload is still a considerable problem with both monopolar and bipolar resection systems (Collins et al. 2005; Hahn 2006).

Although there are some studies in the literature comparing the two techniques, these were mostly conducted by surgical teams, and thus serum sodium levels had not been the primary outcome (Issa 2008; Starkman and Santucci 2005; Engeler et al. 2010; Collins et al. 2005). Previous studies did not include standardization of anesthetic care and iv fluid regimens, and hemodynamic variables were absent too. Thus, the aim of this prospective clinical study was to compare two the different surgical techniques and the systemic effects of irrigation solutions (5 % mannitol vs. 0.9 % sodium chloride) under standardized anesthesia care. Our null hypothesis was that serum sodium levels were the same in both techniques. Our secondary outcomes were hemoglobin levels, heart rate and blood pressures.

This prospective clinical study is registered on ClinicalStudys.gov (NCT02681471) and reported according to the Consolidated Standards of Reporting Trials (CONSORT) statement (Schulz et al. 2010).

## Methods

After obtaining approval by the Institutional Ethics Committee (2012/434-992) and written informed consent from patients for whom TURP operation was planned because of BPH at Istanbul Faculty of Medicine, Istanbul University between December 2013 and April 2015, the patients were enrolled in this study. Patients whose ages ranged between 50 and 90 years and physical status of American Society of Anesthesiology (ASA) class II or III were enrolled to the study. Patients with severe heart failure, respiratory failure, electrolyte imbalance due to neoplasms, diarrhea, vomiting, bleeding diathesis or who refused to join the study or wanted to leave the study were excluded from the study.

Patients were grouped according to the surgeon’s monopolar (group M) or bipolar (group B) resectoscope technique preference. Spinal anesthesia was performed in all patients. An 18G cannula was inserted as venous access and 0.9 % sodium chloride was infused at a rate of 5 mL/kg/h for hydration. Prior to IV route placement (control) and later at the 45th min (1st measurement), and 1 h after the end of the surgery (2nd measurement), 2.5 mL blood samples were drawn from a 16G venous cannula inserted in the antecubital vein of the opposite arm of the patients’ venous access. This blood sample was used to obtain the perioperative serum electrolytes (Na^+^, K^+^). A further 1 mL of blood was collected and placed in a tube with ethylenediaminetetraacetic acid (EDTA) for hemoglobin level evaluation.

Electrocardiogram (ECG), and peripheral oxygen saturation (SpO2) were monitored throughout the anesthesia and non-invasive arterial pressure (NIBP) was measured at 3-min intervals in the first 15 min after spinal anesthesia, and 5-min intervals during rest of the surgery.

Spinal anesthesia was performed in the sitting position from the L4–L5 interspace using a 25G spinal needle with 12.5–15 mg of hyperbaric bupivacaine. The sensory block level was tested to achieve loss of cold sensation at T8–T10 level. The motor block level was also evaluated using a modified Bromage scale (Dar et al. 2015) and surgery was started after establishment of adequate sensory block level. Hypotension was defined as a drop in systolic blood pressure below 30 % of baseline values and was planned to be treated with increasing fluid delivery. If hypotension persisted in the consecutive measurement, 5 mg IV ephedrine was administered. Bradycardia was defined as heart rate <50 beats/min and was planned to be treated with 0.5 mg IV atropine sulfate.

Operations were performed by experienced urology staff. Bipolar TURP was performed using a 24 Fr TURis (OLYMPUS) resectoscope and irrigation fluid containing 0.9 % sodium chloride. Traditional monopolar TURP was performed using a 24 Fr Karl Storz resectoscope and irrigation fluid containing 5 % mannitol (Resectisol Eczacibasi-Baxter). A 22 F three-way Foley catheter was inserted into all patients at the end of surgery and bladder irrigation with normal saline was continued. Patients were admitted to the PACU from the operation room and discharged to the urology ward when discharge criteria were met.

Demographic data of the patients, co-morbidities, preoperative creatinine levels, preoperative prostate volumes determined using transrectal ultrasound (mL), amount of resected prostate tissue (grams) during operation, and duration of the operation were recorded. Hemodynamic data, amount of intravenous fluids administered to patients, volume of irrigation fluids used during operation, and patients requiring vasopressor agent were also recorded. Serum electrolytes (Na^+^, K^+^) and hemoglobin levels were analyzed and recorded. TUR syndrome was defined as perioperative decrease in serum sodium levels (<125 mmol/L) together with at least two of the following symptoms: nausea, vomiting, bradycardia, hypotension, angina, anxiety or confusion (Yousef et al. 2010).

### Statistical analysis

We calculated a priori that we would need 17 persons per group to detect 5 % change in serum Na^+^ levels with α and β errors of 0.05 and 0.2. To allow for withdrawals, we included 26 patients per group.

Data are presented as the mean ± SD or number (% rate). SPSS for Windows version 16.0 was used for statistical analysis. Student’s *t* test was used to analyze quantitative data with parametric distribution. One-way ANOVA was used to evaluate time dependent variations, whereas qualitative data were compared using the Chi square test. *p* < 0.05 was considered statistically significant.

## Results

**Fig. 1:**
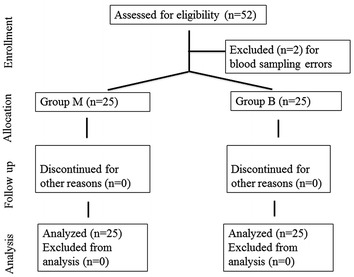
Consolidated Standards of Reporting Trials (CONSORT) diagram of Groups M and B. Group M includes patients undergoing TURP (5 % mannitol was used for irrigation); Group B includes patients undergoing TURP (0.9 % sodium chloride was used for irrigation)

Fifty-two patients who underwent TURP surgery were included in the study. One patient from each group was excluded due to blood sampling errors. Twenty-five patients from each group were evaluated in the final statistical analysis (Fig. 1).

There was no significant difference in age, weight, functional capacity, prostate volume, preoperative levels of creatinine between two groups (Table 1).

**Table 1:** Demographic data

	Group M (n = 25)	Group B (n = 25)	*p*
Age (year)	68.5 ± 8.2	67.8 ± 8.6	0.79^α^
Weight (kg)	75.8 ± 8.4	77.6 ± 10.1	0.48^α^
ASA Class III n, (%)	10 (40)	11(48)	0.56^+^
Co-morbidities n, (%)	18 (72)	17(68)	0.75^+^
Chronic medication use n, (%)	20 (80)	17 (68)	0.33^+^
Prostate volume (ml)	67 ± 26.7	68 ± 29.5	0.87^α^
Preoperative creatinine level (mg/dl)	0.9 ± 0.2	1.14 ± 0.42	0.09^α^
Duration of operation (min)	72 ± 30.9	73 ± 16	0.86^α^

Group M: monopolar, Group B: bipolar

^α^Student’s *t* test

^+^χ^2^ test

**Fig. 2:**
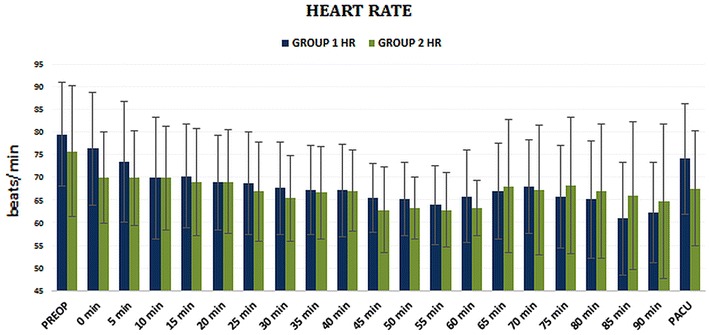
Heart rate (HR) follow-up in Groups 1 and 2 though the perioperative period

**Fig. 3:**
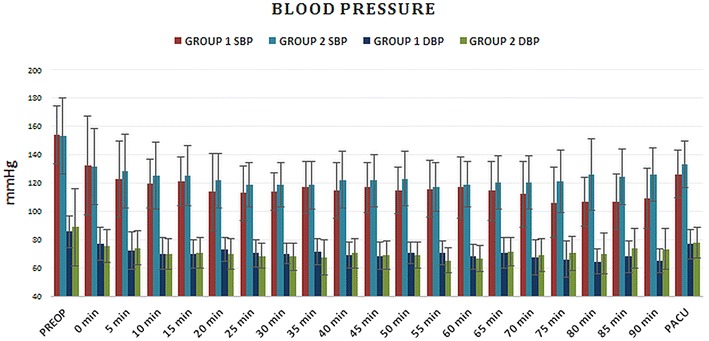
Systolic (SBP) and diastolic blood pressure (DBP) follow-up in Groups 1 and 2 though the perioperative period

Heart rates, systolic and diastolic blood pressures did not differ between the groups at any given time point (Figs. 2, 3). Intravenous fluid administration, frequency of prostatic capsule perforation, and ephedrine administration were also similar between groups (Table 2).

**Table 2:** Patient parameters related surgical and anesthetic data

	Group M (n = 25)	Group B (n = 25)	*p*
IV fluid (ml)	1566 ± 391	1620 ± 661	0.9^α^
Irrigation fluid (ml)	25,360 ± 13,443	27,080 ± 16,240	0.68^α^
Capsule perforation n, (%)	12 (48 %)	13 (52 %)	0.7^+^
Resected prostate weight (g)	49.2 ± 29.8	47.5 ± 13.8	0.83^α^
Ephedrine administration n, (%)	7 (28 %)	5 (20 %)	0.52^+^

^α^Student’s *t* test

^+^χ^2^ test

Volumes of irrigation fluid used were 25,360 ± 13,443 and 27,080 ± 16,240 ml in Groups M and B respectively. Resected prostatic tissue weight were 49.2 ± 29.8 g in Group M and 47.5 ± 13.8 g in Group B (*p* > 0.05) (Table 2) and duration of operations were 72 ± 30.9 and 73 ± 16 in Groups M and B respectively (*p* > 0.05) (Table 1).

There were no statistically significant differences in levels of hemoglobin and K^+^ between and within the groups. Na^+^ value of group M was significantly lower in both the 1st and 2nd measurements compared with the control value (*p* < 0.001 and *p* < 0.001). Na^+^ levels did not differ between groups in control measurement but it was significantly lower in group M than group B in the 1st and 2nd measurements (136.9 vs. 141.8 mmol/L, *p* < 0.001; and 132.7 vs. 140.8 mmol/L, *p* < 0.001 respectively). The change in Na^+^ levels was found to be statistically significant (*p* < 0.001) in group M, whereas the intergroup changes were not statistically significant in group B (Table 3).

**Table 3:** Perioperative sodium, potassium and Hb values of patients

	Group	Control	1st Measurement	2nd Measurement	ANOVA *p*
Hb (g/dL)	M	14.42 ± 2.27	13.5 ± 1.9	13.6 ± 2.4	0.44
	B	13.5 ± 2.67	12.32 ± 2.5	12.33 ± 2.4	0.16
Na^+^ (mmol/L)	M	139.84 ± 3.07	136.9 ± 4.03*	132.68 ± 7.64*	0.001
	B	141.04 ± 3.36	141.8 ± 3.85^+^	140.76 ± 2.73^+^	0.59
K^+^ (mmol/L)	M	3.88 ± 0.47	3.9 ± 0.42	4.06 ± 0.18	0.87
	B	3.7 ± 0.48	3.67 ± 0.41	3.79 ± 0.97	0.82

^α^Student’s *t* test

^β^One Way Anova test

* *p* < 0.001 when compared with control

^+^ *p* < 0.001 when compared with Group M

Two patients in group M required blood because they had Hb levels under 8 g/dL. TUR syndrome was diagnosed in two patients in the monopolar group (8 % incidence) with the lowest serum Na^+^ levels as 111 and 119 mmol/L.

## Discussion

In this prospective study, monopolar resection was associated with lower plasma sodium levels when compared with bipolar resection. Contrary to the monopolar technique, bipolar resection seems to be safe even in patients with extremely high prostate tissue resection and long operation times.

 Monopolar resection, which remains a popular choice for PBH, has been regarded safe according to long-term patient follow-ups but can cause acute complications such as electrolyte imbalances, bleeding, and urethral strictures (Reich et al. 2006; Rassweiler et al. 2006). Electrolyte imbalance is primarily a consequence of systemic absorption of irrigation fluids. In the past, distilled water was used, and was later replaced by other fluids such as glycine 1.2–1.5 %, sorbitol 3 %, urea 1 %, and mannitol 3–5 %. These iso-osmolar fluids overcame the hypo-osmolarity problem in patients undergoing TURP but hyponatremia continued to be the limiting factor in these operations, which result in TUR syndrome when levels under 125 mmol/L are reached.

The discovery of bipolar technology, which is celebrated in urological literature as one of the greatest inventions of the 21st century, enabled the use of normal saline and enhanced surgical view, coagulation, and cauterization (Issa et al. 2004). TUR syndrome has rarely been encountered in recent years since the advent of bipolar technology. Several surgical studies by urologists have compared these two techniques in terms of view, bleeding, and complications (Engeler et al. 2010; Singh et al. 2005; Michielsen et al. 2010; Xie et al. 2012; Hawary et al. 2009; Ho et al. 2007). To our knowledge, the present study with serum electrolytes as a primary outcome, with a standardized anesthesia regimen, and recorded hemodynamic data is the first to compare the effects of 5 % mannitol used in the monopolar technique and normal saline solution, which is used in bipolar resection.

We could not detect a difference in factors other than the type of irrigation fluids that may influence serum electrolytes in our patients. Several factors such as age, functional capacity, co-morbidities, preoperative plasma electrolyte levels, preoperatively determined prostatic weight, resected prostatic volume, resection time, incidence of prostatic capsule perforation, and volume of fluids used for irrigation were found similar between the groups.

We used spinal anesthesia in our patients because it is the most preferred anesthesia method during TURP procedures. Central nervous system symptoms such as restlessness, agitation, confusion, and respiratory system symptoms such as dyspnea, and cyanosis related to TUR syndrome can occur at earlier stages under spinal anesthesia. Blood loss and thromboembolic events are also less common with spinal anesthesia (Singh et al. 2005; Hawary et al. 2009). However, spinal anesthesia can also induce cause hemodynamic disturbances due to symphatectomy and may necessitate fluid administration, which can further influence fluid and electrolyte status of the patients. In our study, blood pressure declined from baseline in both groups but we found no significant difference in blood pressure measurements between the two groups. Similarly, HR was also decreased in both groups after spinal anesthesia as expected. Likewise, intravenous fluid consumption and vasopressor requirements were similar in the study groups. Interestingly, we also did not encounter any low HR between the groups although one would expect hypervolemia to be associated with further HR decrease.

It is well accepted that a considerable amount of irrigation fluid is absorbed into the systemic circulation via prostatic sinuses and perivascular areas in almost all TURP cases. Absorption of 1 L irrigation fluid into the systemic circulation in an hour naturally makes an acute decrease in serum Na^+^ concentrations causing dilutional hyponatremia (Hawary et al. 2009). In our study, we used 5 % mannitol as an irrigation fluid in the monopolar group and found plasma sodium levels significantly lower in the 1st and 2nd measurements when compared with the bipolar group in which isotonic saline was used as irrigation fluid. This reduction in plasma sodium levels has also been reported in other studies that investigated monopolar TURP (Singh et al. 2005; Michielsen et al. 2010). In the present study, 7 mmol/L reductions in plasma sodium concentration is quite striking and is one of the greatest falls in sodium in the literature. We believe that this pronounced decrease in our patients was related to high volumes of prostatic resection and long duration of surgery. Singh et al. found 4.6 meq/L drops in Na concentrations in their monopolar group. In that study, the mean resected prostatic weight was smaller (27 vs. 48 g) and the mean operation duration was shorter (33 vs. 72 min) than in our study (Singh et al. 2005). Interestingly, the closest results to our study were reported by Ho et al. who found 10.7 meq/L of serum sodium reduction with a mean resected prostatic tissue weight of 30 g and resection time of 58 min (Ho et al. 2007).

Parallel to our findings, several authors indicated that plasma Na levels did not demonstrate significant changes during TURP operations performed using a bipolar resection technique. Neyer et al. (2013) and Engeler et al. (2010) reported 0.1 and 0.3 meq/L rise, whereas Singh et al. (2005) and Michielsen et al. (2010) found a 1.2 and 1.5 meq/L drop in sodium concentrations in patients who underwent bipolar TURP. In the present study, it was a striking finding that sodium levels remained unchanged with the bipolar technique, even when patients with high-volume (up to 140 mL) prostates were operated; high volumes of prostate were resected, up to 70 g in TURP operations for as long as 100 min.

Owing to the diluting effects of systemic mannitol absorption, we diagnosed TUR syndrome in two patients in the monopolar group with lowest serum Na^+^ levels of 111 and 119 mmol/L. The patients were discharged to the Urology ward with serum Na^+^ levels higher than 130 mmol/L after 2 days of critical care follow-up. Our results are parallel to a meta-analysis that included 22 clinical trials in which TUR syndrome was solely reported in monopolar-treated group, in 35 of 1375 patients. No TUR syndrome was reported in 1401 patients in the bipolar group (Omar et al. 2014). In this meta-analysis, the highest incidence of TUR syndrome was reported by Yousef et al. (2010) who compared glycine 1.5 %, glucose 5 %, isotonic saline irrigation fluids, and reported TUR syndrome in 17/120 (14 %) patients in the glycine group. Their result was probably correlated with the weight of resected prostatic tissue, which was extremely high with a mean volume of 89 g.

In TURP, perioperative bleeding is one of the most important complications, which leads to clot retention and anemia (Tang et al. 2014). Venous bleeding occurs through open sinuses in TURP operations and perforation of the capsule increases the bleeding (Yousef et al. 2010). Transfusion requirements are reported as 2.9 % in TURP operations, but this incidence increases to 9.5 % when the weight of resected prostatic tissue exceeds 60 g (Reich et al. 2008; Taylor and Jaffe 2015). A meta-analysis of 20 studies that compared monopolar and bipolar methods reported significantly lower incidences of blood transfusion and clot retention in the bipolar group (Omar et al. 2014). The lack of returning current in the bipolar technique provides better hemostasis and minimizes blood loss (Tang et al. 2014). In the present study, the only two patients in group M who developed TUR syndrome had Hb levels under 8 g/dL and required blood transfusion. Despite this reported advantage of bipolar resection in providing hemostasis, we were not able to demonstrate a statistically significant difference in serum Hb levels between the groups. The small amount of blood loss in the monopolar group may be explained by the experience of urologists’ who performed the procedures in our institution.

The limitations of this study were the lack of randomization and reduced number of included subjects (only 25 patients for each arm) although sample size calculated a priori required 17 patients per group. Furthermore, we did not measure 24-h electrolytes as Akçayöz et al. (2006). However, even in their study, the most pronounced changes were found at the end of the operations.

This study implies that using 5 % mannitol is associated with a clinically important decrease in serum Na levels resulting in TUR syndrome, although it doesn’t affect hemodynamic stability.

## Conclusion

Our results confirmed that bipolar resection is much more advantageous in patients undergoing TURP, has minimal effects on serum sodium, and thus minimizes the risk of TUR syndrome compared with monopolar resection.
